# Does use of anal cytology as a triage test improve the performance of high‐risk human papillomavirus screening in gay and bisexual men for anal cancer prevention?

**DOI:** 10.1002/ijc.35185

**Published:** 2024-09-15

**Authors:** Fengyi Jin, I. Mary Poynten, Richard J. Hillman, Carmella Law, Monica Molano, Christopher K. Fairley, Suzanne M. Garland, David J. Templeton, Andrew E. Grulich, Jennifer Roberts, the SPANC study team, Brian Acraman, Andrew Carr, Susan Carroll, David Cooper, Alyssa Cornall, Leonie Crampton, Christopher Fairley, Annabelle Farnsworth, Lance Feeney, Eddie Fraissard, Suzanne Garland, Andrew Grulich, Richard Hillman, Kirsten Howard, Fengyi Jin, Carmella Law, Matthew Law, Dorothy Machalek, Kirsten McCaffery, Patrick McGrath, Robert Mellor, Richard Norris, Matthew O'Dwyer, Susan Pendlebury, Kathy Petoumenos, Samuel Phillips, Monica Molano, I Mary Poynten, Garrett Prestage, Adele Richards, Jennifer Roberts, Lance Schema, Daniel Seeds, Eva Segelov, Sepehr Tabrizi, Dave Templeton, Julia Thurloe, Winnie Tong, Rick Varma

**Affiliations:** ^1^ The Kirby Institute, University of New South Wales Sydney New South Wales Australia; ^2^ Dysplasia and Anal Cancer Services, St Vincent's Hospital Sydney New South Wales Australia; ^3^ Murdoch Children's Research Institute Melbourne Victoria Australia; ^4^ Department of Obstetrics and Gynaecology Centre Women's Infectious Diseases Research, Royal Women's Hospital, University of Melbourne Melbourne Victoria Australia; ^5^ Melbourne Sexual Health Centre, and Central Clinical School, Monash University Melbourne Victoria Australia; ^6^ Department of Sexual Health Medicine Sydney Local Health District Sydney New South Wales Australia; ^7^ Discipline of Medicine, Central Clinical School, Faculty of Medicine and Health, the University of Sydney Sydney New South Wales Australia; ^8^ Douglass Hanly Moir Pathology Sydney New South Wales Australia

**Keywords:** anal cancer, cancer screening, homosexuality, human papillomavirus, male, screening performance

## Abstract

Anal high‐risk human papillomavirus (HRHPV) testing‐based anal cancer screening gay and bisexual men (GBM) is associated with high sensitivity, but low specificity. We report the potential role of triage use of anal cytology with HRHPV testing in detecting 12‐month persistent anal high‐grade squamous epithelial lesions (HSIL) in a cohort of GBM in Sydney, Australia. Participants were GBM from the Study of the Prevention of Anal Cancer (SPANC) who underwent annual anal HPV testing, cytology, and high‐resolution anoscopy (HRA)‐guided histology. The sensitivity and specificity of five screening algorithms based on HRHPV test results with triage use of anal cytology (atypical squamous cells of undetermined significance (ASCUS) and atypical squamous cells, cannot exclude HSIL (ASC‐H) used as referral thresholds) were compared to these of HRHPV testing and anal cytology alone. A total of 475 men who had valid HRHPV, cytological, and histological results at both baseline and first annual follow‐up visits were included, median age 49 years (inter‐quartile range: 43–56) and 173 (36.4%) GBM with human immunodeficiency virus. Of all triage algorithms assessed, two had comparable sensitivity with HRHPV testing alone in detecting persistent anal HSIL, but ~20% higher specificity and 20% lower HRA referral rates. These two algorithms involved the immediate referral of those with HPV16 and for those with non‐16 HRHPV either immediate or delayed (for 12 months) referral, depending on cytology result at baseline. Triage use of anal cytology in GBM testing positive for anal HRHPV increases specificity and reduces referral rates while maintaining high sensitivity in detection of HSIL.

## What's New?

Anal cancer screening in high‐risk groups has been advocated, but evidence for a preferred strategy is lacking. Using data from a 3‐year natural history study of anal HPV infection in gay and bisexual men, the authors calculated the theoretical referral rate for a range of algorithms and the sensitivity and specificity in detecting precursor lesions. Two algorithms using primary HPV testing (with genotyping) and anal cytology as triage showed marked improvements in screening specificity and referral number compared to primary HPV testing alone, while maintaining a high sensitivity. The analysis may guide the design of efficient anal cancer screening programs.

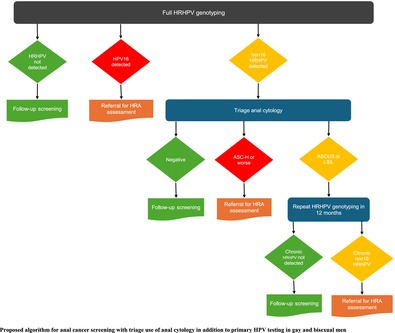

## 1 INTRODUCTION

Anal cancer disproportionately affects gay, bisexual, and other men who have sex men (GBM), particularly, those living with human immunodeficiency virus (HIV).^1^ Persistent infection with high‐risk human papillomavirus (HRHPV) is a necessary cause for anal cancer,^2^ with HPV16 being the leading causal type, detected in ~80% of cases.^3^, ^4^, ^5^ However, HPV16 is less frequently detected in anal cancers in people living with HIV(PLHIV, 67%) than in those without HIV (82%).^4^

Anal cancer screening in high‐risk groups has been advocated, with the aim of detecting and treating the cancer precursor, high‐grade squamous intraepithelial lesion (HSIL), to prevent cancer development,^6^ as is the case for cervical cancer screening. In cervical screening, the use of cytology‐based screening tests has been recently replaced in many countries with primary HPV screening, for its superior sensitivity and objectivity in detecting cervical HSIL.^7^ In some settings where primary HPV cervical screening has been implemented, cytology is now used as a triage test in women who test positive to HRHPV to improve screening specificity^8^ and thereby to determine prioritisation to colposcopy. In Australia, women who test positive for an HRHPV type other than 16 or 18 through primary HPV screening are recommended to undergo triage cytology, and only those with atypical squamous cells, cannot exclude HSIL (ASC‐H) or worse on that test are then referred for immediate colposcopy.^9^

The sensitivities of cytology and HPV testing for detection of anal HSIL are similar to those found in their use in cervical screening.^7^, ^10^ However, HRHPV is so prevalent in GBM, that the use of HRHPV testing as a sole screening test leads to extremely high referral rates and low specificity, despite its excellent sensitivity.^10^ The specificity of anal cytology alone is also low in this population,^10^ due to the presence of many incidental low‐grade non‐neoplastic lesions. There are some differences in HPV‐associated carcinogenesis. First, HPV16 alone causes the majority (~80%) of anal cancer cases,^4^ whereas in cervix HPV16 (~54%) and HPV18 (~13%) are the leading causes.^11^ Second, there is a higher spontaneous clearance of anal HSIL. Lack of clearance is associated with prevalent HPV16 infection or chronic infection of non‐16 HRHPV types.^12^

The Anal Cancer‐HSIL Outcomes Research (ANCHOR) clinical trial demonstrated that HSIL treatment reduced anal cancer risk by nearly 60% in PLHIV,^13^ prompting the release of consensus anal cancer screening guidelines by the International Anal Neoplasia Society.^14^ These guidelines outline various currently practised screening algorithms using combinations of anal cytology and/or HRHPV testing, but acknowledge that a preferred option cannot be recommended, due to the absence of evidence. With respect to HRHPV testing with triage cytology, these guidelines explicitly state that ‘observational data on this approach are lacking in the literature’.^14^

We report on the performance of HRHPV testing with and without use of anal cytology as a triage test and compare these results with cytology and HRHPV screening alone in our three‐year cohort of GBM in Sydney, Australia, who all underwent annual HPV and cytology testing and high‐resolution anoscopy (HRA), a diagnostic procedure analogous to colposcopy in cervical cancer screening. We report results for different screening test result thresholds with respect to referral for HRA. Given the relatively high rate of spontaneous clearance of anal HSIL that we have documented in this population previously,^12^ in this report we use 12‐month persistent histologically diagnosed HSIL as the outcome of interest.

## 2 METHODS

Participants in the Study of the Prevention of Anal Cancer (SPANC) were recruited mainly from community‐based settings in Sydney from September 2010 to August 2015. The methods of the study have been described in detail elsewhere.^15^ Briefly, SPANC was a longitudinal study of the natural history of anal HPV infection. Men aged 35 years and older who reported having sex with another man in their lifetime were eligible. Those who previously had an HRA or a history of anal cancer were excluded.

After study enrolment, participants attended a baseline visit and three scheduled annual follow‐up visits. At each study visit, they underwent anal HPV testing and cytology, and histological assessments guided by HRA.

### 2.1 HPV genotyping

A Dacron® swab moistened with tap water was used to sample the anal canal by a study clinician. Immediately after sampling, the swab was rinsed in a vial containing 20 mL of PreservCyt (Hologic, Inc., Marlborough, MA, USA) fixative medium.

Prior to cytology processing, an aliquot of 4 mL of PreservCyt was removed and forwarded to the Regional HPV Lab Net Reference Laboratory, Melbourne, Victoria, and stored at −80°C. HPV genotyping was performed using Roche Linear Array (LA) HPV genotyping test (Roche Molecular Systems, Alameda, CA, USA) as described previously.^16^ The LA HPV genotyping test allowed for the identification of 37 HPV genotypes. HPV16, 18, 31, 33, 35, 39, 45, 51, 52, 56, 58, 59, and 68 were considered HRHPV.^17^

We have shown that the lower limit of detection of HPV using LA is substantially higher than for the Anyplex™ II HPV HR Detection (Seegene, Seoul, South Korea)^18^ for non‐16 HRHPV types. Therefore, stored ThinPrep residuum was also tested with the Anyplex™ II HPV HR assay. HPV results in the analyses were based on detection of each HPV type by either LA and/or Anyplex™ testing.

### 2.2 Anal cytology

At a specialist anogenital pathology laboratory, a ThinPrep slide (Hologic Inc., Marlborough, MA, USA) was produced and manually screened by an experienced study cytologist. One of the three study pathologists was responsible for final reporting using the Bethesda System (TBS) 2014 criteria and terms.^19^ The cytological results were classified as negative, atypical squamous cells of undetermined significance (ASCUS), low‐grade squamous intraepithelial lesion (LSIL), ASC‐H, or HSIL.

### 2.3 High‐resolution anoscopy

HRA was performed immediately after the anal swab. Biopsies of visual abnormalities suspicious of HPV‐related lesions were taken and placed into a formalin solution for histopathological assessment. Reporting of the biopsies was performed in accordance with criteria, terminology and recommendations of the Lower Anogenital Squamous Terminology (LAST) Project.^20^, ^21^ Results were reported as negative, LSIL, HSIL‐AIN2, or HSIL‐AIN3. As recommended by LAST, p16 stain was used to confirm all HSIL‐AIN2 diagnoses and to distinguish between HSIL and benign mimics when required. The highest‐grade abnormality was used for the analysis. Biopsies were not taken in participants who had no visual abnormalities suggestive of HPV‐related lesions on HRA examination and these participants were classified as negative for SIL, as in our previously published analysis.^22^

### 2.4 Choice of theoretical thresholds for referral to HRA

Among men who had valid results at all visits for HPV, cytology, HRA appearance, and histological results on biopsy at HRA, we examined three different theoretical approaches for anal cancer screening: HRHPV testing alone, anal cytology alone, and the triage use of anal cytology based on HRHPV test results.

#### 2.4.1 Theoretical HRA referral thresholds based on anal HRHPV testing alone

We first assessed the screening performance in those who tested positive to any HRHPV at baseline.

Then we examined a two‐tier referral schedule based on HRHPV test results. First, a positive baseline test for HPV16 met the threshold for immediate referral for HRA. Second, for men who tested positive only for non‐16 HRHPV at baseline results from the 12 months anal swab were reviewed. Only those who had the same HRHPV type detected at both visits (demonstrating likely chronic HRHPV infection), met the threshold for referral for HRA. The rationale for this approach is that HPV16 causes the majority (~80%) of anal cancer cases^4^ and that lack of clearance of HRHPV is the major risk factor for HSIL development and subsequent carcinoma, hence the need to discriminate between transient and chronic HPV infection.

#### 2.4.2 Theoretical HRA referral thresholds based on anal cytology alone

Participants who had any cytological abnormality (ASCUS or worse) met the theoretical referral threshold. Additionally, we also assessed the screening performance when ASC‐H or worse was used as the referral threshold.

#### 2.4.3 Theoretical HRA referral thresholds based on anal HRHPV‐testing with triage to cytology in those testing HRHPV positive

**FIGURE 1:**
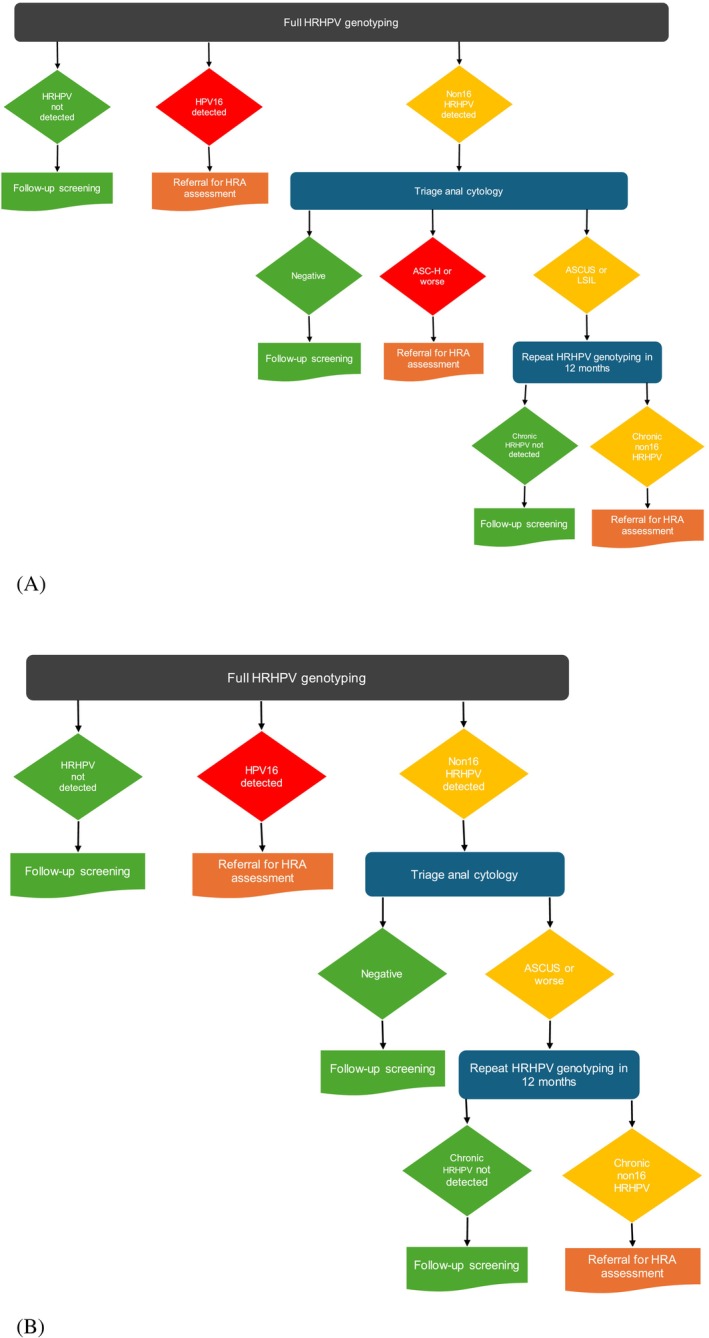
High‐resolution anoscopy referral (A, Algorithm 1; B, Algorithm 2) pathway based on high‐risk human papillomavirus (HRHPV) testing with cytology as triage test.

For men who tested HRHPV positive at baseline, we developed two algorithms, with respect to varying HRHPV and cytology test results, loosely based on algorithms previously developed for the Australian cervical screening program.^9^ In both algorithms, given the more pathogenic effect of HPV16, those who tested positive for HPV16 met the HRA threshold regardless of cytology results (although cytology may be used to provide additional information for the referral anoscopist). In Algorithm 1 (Figure 1A), in those with non‐16 HRHPV at baseline, given the high positive predictive value of ASC‐H cytology,^23^ this was the threshold for immediate HRA. For those with lesser cytological abnormality, evidence of 12‐month persistence of the non‐16 HRHPV genotype was required prior to referral for HRA. In Algorithm 2 (Figure 1B), in those with non‐16 HRHPV at baseline, the threshold for HRA referral was ASCUS+ cytology at baseline and persistence of the non‐16 HRHPV genotype at 12 months. Three additional algorithms (Algorithms 3–5, Table 1) aiming for higher screening specificity were developed to assess its impact on overall screening performance.

**TABLE 1:** Additional algorithms aiming for higher specificity in triage use of anal cytology in men testing positive to HRHPV at baseline in predicting 12‐month persistent anal high‐grade squamous intraepithelial lesions.

		Baseline screening results			12‐Month screening results
		HPV16	Non‐16 HRHPV	Cytology	Non‐16 HRHPVgenotype
Algorithm 3: Refer for HRA if	Either	Pos		No cyto triage	
	Or	Neg	Pos	ASC‐H+	Persistent
Algorithm 4: Refer for HRA if	Either	Pos		ASCUS+	
	Or	Neg	Pos	ASCUS+	Persistent
Algorithm 5: Refer for HRA if	Either	Pos		ASC‐H+	
	Or	Neg	Pos	ASC‐H+	Persistent

*Note*: ASCUS, atypical squamous cells of undermined significance; Persistent refers to type‐specific non‐16 high‐risk HPV infection detected at both baseline and 12‐month visits.

Abbreviations: ASC‐H, atypical squamous cells, cannot exclude high‐grade squamous intraepithelial lesions; ASCUS, atypical squamous cells of undetermined significance; HPV, human papillomavirus; HRA, high‐resolution anoscopy; HRHPV, high‐risk human papillomavirus.

### 2.5 Disease endpoint‐persistent histological anal HSIL

Persistent anal HSIL in a participant was defined as histologically confirmed anal HSIL (including HSIL‐AIN2 and HSIL‐AIN3) at both baseline and the first follow‐up study visit.

### 2.6 Statistical analysis

Statistical analyses were performed using Stata 18.0 (Stata Corporation, College Station, TX, USA). Characteristics of participants who had all test results available and thus could be included in the analysis were compared to those who were not, using chi‐square tests.

Theoretical HRA referral rates were calculated, as described above. We then calculated sensitivity and specificity for the detection of 12‐month persistent anal HSIL. For each screening test and algorithm assessed, analyses were then repeated stratified by HIV status, and sensitivities and specificities by HIV status were compared using Chi‐square tests.

## 3 RESULTS

Between 2010 and 2015, a total of 617 participants were recruited. Among them, 475 (77.0%) attended at least one annual follow‐up visit, had valid test results available for each of anal cytology, HPV genotyping, and HRA, including histological assessment where there were any visible abnormalities at both baseline and first annual visits. These 475 participants were included in the analysis. The median age was 49 years (inter‐quartile range: 43–56), and 173 (36.4%) were GBM with HIV (Table 2). Participants who were included in the analysis (*n* = 475) were broadly similar to those who were not (*n* = 142) in terms of age, HIV status, and number of recent (last 6 months) and lifetime sexual partners. The exception was that those included in the analysis were less likely to be past or current cigarette smokers (*p* = .002, Table 2).

**TABLE 2:** Characteristics of participants who were included and not included in the analysis at baseline.

	Participants included in the analysis				*p‐*Value
	Yes (*N* = 475)		No (*N* = 142)		
	*n*	%	*n*	%	
Age					.414
35–44	146	30.7	52	36.6	
45–54	186	39.2	50	35.2	
≥55	143	30.1	40	28.2	
HIV status					.568
GBM without HIV	302	63.6	94	66.2	
GBM with HIV	173	36.4	48	33.8	
Ever cigarette smoking					.002
Never	275	57.9	59	41.6	
Post	141	29.7	55	38.7	
Current	59	12.4	28	19.7	
Lifetime number of sexual partners					.318
<10	6	1.3	6	3.7	
11–50	80	17.1	22	16.1	
51–200	136	29.1	37	27.0	
201–500	90	19.3	32	23.4	
>500	155	33.2	41	29.9	
Number of sexual partners in the last 6 months					.104
None	32	6.7	17	12.0	
1	93	19.6	30	21.1	
2–5	126	26.5	38	26.8	
6–10	79	16.6	27	19.0	
>10	145	30.5	30	21.1	
Anal cytology					.319
Normal	190	40.0	51	45.1	
Abnormal	285	60.0	62	54.9	
Anal histology					.210
No HSIL	318	67.0	103	72.5	
HSIL	157	33.1	39	27.5	
Anal HPV					.507
HRHPV negative	106	22.3	38	27.0	
Non‐16 HRHPV	209	44.0	57	40.4	
HPV16	160	33.7	46	32.6	

Abbreviations: HRHPV, high‐risk human papillomavirus; HSIL, high‐grade squamous intraepithelial lesion.

Of the 475 men included in the analysis, 439 attended the first 12‐month annual follow‐up visit. For an additional 36 men, as their first annual follow‐up visit was missed, we included data from their 24‐month (*n* = 28) or 36‐month study visit (*n* = 8).

### 3.1 Disease outcome

At baseline, 157 men (33.1%) had histologically confirmed anal HSIL and of these persistent HSIL at the follow‐up visit was found in 88 (56.1%).

### 3.2 Theoretical HRA referral thresholds for HRA based on anal HRHPV testing alone

At baseline, 160 (*n* = 33.7%) men tested HPV16 positive. An additional 209 (44.0%) tested positive to at least one non‐16 HRHPV type, among whom, evidence of persistent type‐specific non‐16 HRHPV infection was found in 167 (79.9%) at their first annual follow‐up. Among these 167 men, persistent infection with HPV18 was most frequent (*n* = 39, 23.4%), followed by HPV58 (*n* = 36, 21.6%), HPV68 (*n* = 35, 21.0%), HPV52 (*n* = 26, 15.6%), and HPV39 (*n* = 23, 13.8%).

Using detection of any HRHPV at baseline as a screening test, the sensitivity and specificity for detection of persistent anal HSIL were 100.0% and 27.4%, respectively, with 77.7% of men meeting the HRA referral threshold (Table 3). Considering those who tested HPV16 positive at baseline or had persistent non‐16 HRHPV infection for HRA referral, the sensitivity remained very high (97.7%) and the specificity increased to 37.7%, with about 10% fewer (68.8%) meeting the HRA referral threshold (Table 3).

**TABLE 3:** HPV and anal cytology screening test performance in predicting persistent 12‐month anal high‐grade squamous intraepithelial lesion, stratified by HIV status.

	Screen positive		Sensitivity		Specificity	
	*n* (%)	95% CI	%	95% CI	%	95% CI
*HPV testing‐based screening*						
Any HRHPV at baseline	369 (77.7)	73.7–81.4	100.0	95.9–100.0	27.4	23.0–32.1
*By HIV status*						
GBM without HIV	73.8	68.5–78.7	100.0	91.8–100.0	30.5	25.0–36.5
GBM with HIV	84.3	78.1–89.4	100.0	92.1–100.0	21.1	14.4–29.2
HPV16 positive at baseline or persistent non‐16 HRHPV^a^	327 (68.8)	64.5–73.0	97.7	92.0–99.7	37.7	32.9–42.8
*By HIV status*						
GBM without HIV	64.6	58.9–70.0	100.0	91.8–100.0	41.3	35.3–47.6
GBM with HIV	76.3	69.3–82.4	95.6	84.9–99.5	30.5	22.6–39.2
*Cytology‐based screening*						
ASCUS as cut‐off	285 (60.0)	55.4–64.4	89.8	81.5–95.2	46.8	41.7–51.9
*By HIV status*						
GBM without HIV	56.3	50.5–62.0	88.4	74.9–96.1	49.0	42.8–55.3
GBM with HIV	66.5	58.9–73.5	91.1	78.8–97.5	42.2	33.5–51.2
ASC‐H as cut‐off	159 (33.5)	29.2–37.9	70.5	59.8–79.7	74.9	70.3–79.2
*By HIV status*						
GBM without HIV	34.4	29.1–40.1	76.7	61.4–88.2	72.6	66.7–77.9
GBM with HIV	31.8	24.9–39.3	64.4	48.8–78.1	79.7	71.7–86.3

Abbreviations: ASC‐H: atypical squamous cells, cannot exclude high‐grade intraepithelial lesions; ASCUS, atypical squamous cells of undetermined significance; GBM, gay and bisexual men; HIV, human immunodeficiency virus; HPV, human papillomavirus; HRHPV, high‐risk human papillomavirus.

^a^ Participants who: (1) tested HPV16 positive at baseline or (2) tested negative to HPV16 at baseline but had evidence of persistent type‐specific non‐16 HRHPV at 12‐months.

### 3.3 Theoretical HRA referral thresholds based on anal cytology alone

Overall, the standalone anal cytology‐based screening using ASCUS or worse as the referral threshold had a sensitivity of 89.8% and a specificity of 46.8% in detection of persistent anal HSIL, with a theoretical referral rate of 60.0% (Table 3). When ASC‐H or worse was used as the referral threshold, the sensitivity was lower (70.5%) and the specificity higher (74.9%), with a much lower referral rate of 33.5% (Table 3).

### 3.4 Theoretical HRA referral thresholds based on anal HRHPV‐testing with triage to cytology in those testing HRHPV positive

The performance of triage use of anal cytology was evaluated according to the algorithms outlined in Table 1.

Using Algorithm 1, those who tested HPV16 positive at baseline were referred regardless of anal cytology status. Those who had tested non‐16 HRHPV at baseline were only referred if they also had ASC‐H or worse cytology at baseline or if they had evidence of persistent non‐16 HRHPV infection at 12 months and had ASCUS or worse cytology at baseline. Under this scenario, the sensitivity was 95.5%, and the specificity was 49.1%, with a theoretical HRA referral rate of 59.2% (Table 4).

**TABLE 4:** Screening test performance in five different algorithms applying triage use of cytology testing in men who screen positive for HRHPV in predicting persistent 12‐month anal high‐grade squamous intraepithelial lesion, stratified by HIV status.

	Screen positive		Sensitivity		Specificity	
	*n* (%)	95% CI	%	95% CI	%	95% CI
Algorithm 1^a^	281 (59.2)	54.6–63.6	95.5	88.8–98.7	49.1	44.0–54.2
*By HIV status*						
GBM without HIV	55.3	49.5–61.0	97.7	87.7–99.9	51.7	45.5–58.0
GBM with HIV	65.9	58.3–72.9	93.3	81.7–98.6	43.8	35.0–52.8
Algorithm 2^b^	273 (57.5)	52.8–62.0	94.3	87.2–98.1	50.9	45.8–56.0
*By HIV status*						
GBM without HIV	53.0	47.2–58.7	97.7	87.7–99.9	54.4	48.2–60.6
GBM with HIV	65.3	57.7–72.4	91.1	78.8–97.5	43.8	35.0–52.8
Algorithm 3^c^	215 (45.3)	40.7–49.9	80.7	70.9–88.3	62.8	57.8–67.6
*By HIV status*						
GBM without HIV	44.0	38.4–49.8	88.4	74.9–96.1	63.3	57.1–69.2
GBM with HIV	47.4	39.8–55.1	73.3	58.1–85.4	61.7	52.7–70.2
Algorithm 4^d^	235 (49.5)	44.9–54.1	87.5	78.7–93.6	59.2	54.1–64.1
*By HIV status*						
GBM without HIV	45.0	39.3–50.8	88.4	74.9–96.1	62.2	56.0–68.1
GBM with HIV	57.2	49.5–64.7	86.7	73.2–94.9	53.1	44.1–62.0
Algorithm 5^e^	142 (29.9)	25.8–34.2	69.3	58.6–78.7	79.1	74.7–83.0
*By HIV status*						
GBM without HIV	29.5	24.4–35.0	76.7	61.4–88.2	78.4	72.9–83.2
GBM with HIV	30.6	23.9–38.0	62.2	46.5–76.2	80.5	72.5–86.9

Abbreviations: ASC‐H, atypical squamous cells, cannot exclude high‐grade squamous intraepithelial lesion; ASCUS, atypical squamous cells of undetermined significance; GBM, gay and bisexual men; HIV, human immunodeficiency virus; HPV, human papillomavirus; HRHPV, high‐risk human papillomavirus.

^a^ Screening positive: Participants who: (a) tested HPV16 positive at baseline regardless of cytology status or (b) tested non‐16HRHPV positive at baseline and ASC‐H or worse cytology at baseline or (c) tested negative to HPV16 at baseline but had evidence of persistent type‐specific non‐16 HRHPV at 12 months AND ASCUS or worse cytology at baseline.

^b^ Screening positive: Participants who: (a) tested HPV16 positive at baseline regardless of cytological status or (b) tested negative to HPV16 at baseline but had evidence of persistent type‐specific non‐16HRHPV at 12 months AND ASCUS or worse cytology at baseline.

^c^ Screening positive: Participants who: (a) tested HPV16 positive at baseline regardless of cytological status or (b) tested negative to HPV16 at baseline but had evidence of persistent type‐specific non‐16HRHPV at 12 months AND ASC‐H or worse cytology at baseline.

^d^ Screening positive: Participants who: (a) tested HPV16 positive AND had ASCUS or worse cytology at baseline or (b) tested negative to HPV16 at baseline but had evidence of persistent type‐specific non‐16HRHPV at 12 months AND ASCUS or worse cytology at baseline.

^e^ Screening positive: Participants who: a. tested HPV16 positive AND had ASC‐H or worse cytology at baseline or (b) tested negative to HPV16 at baseline but had evidence of persistent type‐specific non‐16HRHPV at 12 months AND ASC‐H or worse cytology at baseline.

Using Algorithm 2, those who tested HPV16 positive at baseline were referred regardless of cytology status, and those who had persistent non‐16 HRHPV were only referred if there was ASCUS or worse anal cytology present at baseline. This approach had a sensitivity of 94.3% and a specificity of 50.9%, with a referral rate of 57.5% (Table 4).

Using Algorithm 3, those who tested HPV16 positive at baseline were referred regardless of anal cytology status, and those had persistent non‐16 HRHPV were only referred if they had ASC‐H or worse cytology at baseline. Under this scenario, the sensitivity was 80.7% and the specificity was 62.8%, with a referral rate of 45.3% (Table 4).

Using Algorithm 4, those who had ASCUS or worse anal cytology at baseline and tested HPV16 positive at baseline or persistent non‐16 HRHPV at first annual follow‐up were referred. Under this scenario, the sensitivity was 87.5% and the specificity was 59.2%, with a referral rate of 49.5% (Table 4).

Using Algorithm 5, those who had ASC‐H or worse anal cytology at baseline and tested HPV16 positive at baseline or persistent non‐16 HRHPV at follow‐up were referred. Under this scenario, the sensitivity was lower at 69.3% and the specificity was higher at 79.1%, with a lower referral rate of 29.9% (Table 3).

### 3.5 Screening performance by HIV status

In screening tests using HRHPV testing alone, there were no differences in screening sensitivity between GBM with and without HIV, but specificity was higher in GBM without HIV in both scenarios (*p* = .048 and *p* = .037 for using any HRHPV and HPV16/persistent non‐16 HRHPV as referral thresholds, respectively). There were no differences in screening performance with cytology alone regardless of the referral threshold.

In screening tests using anal cytology as triage tests, there were no differences by HIV status in screening performance in Algorithms 1, 3, 4, and 5. There was higher screening specificity in GBM without HIV than those with HIV for Algorithm 2 (*p* = .048).

## 4 DISCUSSION

Treating anal HSIL has been proven to prevent cancer in those PLHIV,^13^ and there is a need for accurate and reliable screening tests for detection of anal HSIL in those at high risk. In this cohort study, we have demonstrated that the use of anal cytology as a triage test can improve the specificity of HRHPV testing in identifying those anal HSIL lesions in need of treatment. Among GBM in Sydney, we have shown that HRHPV testing alone was highly sensitive in detecting persistent anal HSIL, ~10% higher than that of anal cytology alone, but with inferior specificity. This could prevent the use of HRHPV testing as a standalone test for anal cancer screening. Two of the theoretical algorithms with the triage use of cytology that we developed (Algorithms 1 and 2) were effective in reducing referral rates to HRA by ~20% compared to HRHPV testing alone (59.2% or 57.5% vs. 77.7%, respectively). There was only ~ 5% reduction in sensitivity (95.5% or 94.3% vs. 100.0%, respectively) and an increase in specificity of more than 20% (49.1% or 50.9% vs. 27.4%, respectively).

Anal cancer screening has been modelled on cervical cancer screening, which has a long history of success in reducing the incidence of cervical cancer,^24^, ^25^ firstly with cytology‐based screening and more recently with primary HPV testing for its superior sensitivity in detection of cervical HSIL.^7^ The shift to primary HPV screening in the cervix was informed by numerous large‐scale randomised controlled trials and cohort studies.^26^ However, improvements in the sensitivity were accompanied by initial increases in colposcopy referral and false positive rates.^26^ For a cancer screening program aimed at the general population, a high specificity must be maintained because the majority of those of screening age will not have the precancerous condition. A small decrease in specificity may result in thousands of those screened undergoing unnecessary referral diagnostic procedures, adding to the pressure on public health resources and personal anxiety. To overcome this decreased specificity in using primary HRHPV testing, many European countries and Australia, have retained cervical cytology as a triage test in those who test positive to HRHPV in primary HRHPV screening programs to reduce the rates of false positive and colposcopy referral.^9^, ^26^, ^27^

Compared to the cervix, anal cytology has a similar high sensitivity in detection of anal HSIL in GBM with a pooled estimate of 90.8%, but a much poorer specificity of 35.5%.^28^ Systematic reviews have reported anal HRHPV testing also has a high sensitivity of 91.3% (close to 100% in our study), but a low specificity of 33.1%.^10^, ^29^ The low specificity of anal cytology and HRHPV testing has hampered their utility as primary screening tests for anal cancer prevention. This prompted us to develop a two‐tier screening strategy based on HRHPV test results, similar but not identical to the cervical screening model. Based on our findings, we propose first that participants testing positive for HPV16 be directly referred for HRA.^4^ Second, for participants positive for non‐16 HRHPV genotypes, we propose that persistence for a 12‐month period must be established prior to HRA referral in the absence of ASC‐H or worse cytology as we have shown that transient HRHPV infection is associated with higher rates of spontaneous anal HSIL clearance.^12^ The triage use of cytology after HRHPV testing, particularly in the presence of non‐16/18 HRHPV, has been supported by longitudinal studies in the field of cervical research for improvements in specificity,^30^, ^31^, ^32^, ^33^ but research on its use in anal cancer screening is very limited.

An anal cancer screening program that has a 12‐month waiting period for further action will have implication for screening adherence, which is vital for its success in cancer prevention. Should similar anal cancer screening programs be recommended, the importance of adherence should be emphasised based on their initial screening results, that is, in those who test positive to non‐16 HRHPV and have abnormal anal cytology, to ensure maximum retention for the recommended 12‐month screening regime. An effective recall system coordinated by centralised screening registers, similar to the Australian National Cancer Screening Register,^34^ is also critical to ensure these GBM are compliant with the screening recommendations.

The two screening algorithms that we assessed, Algorithms 1 and 2, have very similar performance characteristics. Nevertheless, Algorithm 1 has the additional advantage of immediately referring those with ASC‐H+ cytology to HRA if they test positive to non‐16 HRHPV, without the need for a 12‐month wait to establish persistent non‐16 HRHPV infection, and thus is preferred in our opinion. This is due to the high positive predictive value for anal histological HSIL associated with ASC‐H+ cytology as we reported previously.^23^ The adoption of either, however, could be up to individual practices' preference and according to their patients' characteristics. Algorithm 1 might be more suitable for GBM with HIV because of a higher proportion of anal cancer attributable to non‐16 HRHPV or because of patient anxiety or clinician concern regarding loss to follow‐up. On the other hand, Algorithm 2 only refers those with HPV16 immediately to HRA, which might be better suited to GBM without HIV, in whom the proportion of anal cancer caused by HPV16 is higher.^4^

There are also cancer risk concerns in those who are subject to the 12‐month wait, that is, those screened non‐16 HRHPV positive. Primary HPV screening and anal cytology should be performed as a part of comprehensive cancer prevention measures in high‐risk populations, which should also include anal digital anal rectal examination,^35^ for detection of early‐stage anal cancer which is palpable. The proposed screening algorithms would capture those who are HPV16 positive for immediate HRA referral, and additionally, those with ASC‐H+ anal cytology subject to patient characteristics and HRA capacity. The risk of anal cancer remains unknown in those screened positive to non‐16 HRHPV. While a high progression rate has been reported in the ANCHOR study in participants of the monitoring arm, the evidence is lacking on the progression rate in those who are absent of HPV16 and ASC‐H^13^.

Persistent HSIL is the necessary precursor for HPV‐associated anal and cervical cancers. In cervical cancer screening, there is less emphasis on establishing HSIL persistence because of the higher risk of cervical HSIL progressing to cancer than that of anal HSIL^36^ and because curative treatment strategies have been available for decades. In a natural history study of anal HSIL in GBM, the estimated progression rate from anal HSIL to cancer is much lower, at 0.27% and 0.02% per annum, respectively, in GBM with and without HIV,^37^ and spontaneous HSIL clearance is common.^12^ The ANCHOR study, published in 2022, reported a higher progression rate of 0.41% per annum in their PLHIV participants assigned to the active monitoring arm.^13^ At either progression rate, it is clear that anal HSIL treatment is warranted to prevent development of anal cancer in high‐risk populations like PLHIV.

The majority of the SPANC study participants were recruited from community‐based settings in Sydney, Australia, and thus one of the main strengths of the present study is that screening performance characteristics are likely to be representative of community‐attached GBM. As a natural history study of anal HPV infection conducted prior to the ANCHOR study results were available, participants diagnosed with histological anal HSIL were subject to close monitoring, rather than active treatment. Thus, SPANC is one of the few studies that is capable of assessing anal cancer screening modalities with persistent HSIL as the disease endpoint. Further screening algorithms can be assessed. In the SPANC study, the use of multiple HPV testing assays maximised the sensitivity in detection of non‐16 HRHPV to more accurately assess persistent status of those HPV genotypes.^38^ However, the proportion of GBM with non‐16 HRHPV persistence may differ in settings where different HPV testing assays are used. Conventionally, LA was only used in research settings, and its production has now been ceased by the manufacturer.^38^ A list of other commercially available HPV assays has been validated for primary cervical cancer screening, including AnyPlex II that was used in the current study.^39^ Cytology is a field in which extensive training and experience are required for high performance. Its utility in cancer screening is usually confined to industrialised countries. The cytologists and histopathologists associated with the study were from the largest private anogenital pathology service in Australia with high inter‐observer reproducibility.^40^ The possibility of triage use of anal cytology in additional to HPV testing as screening tests for anal cancer prevention might not be viable in resource‐limited settings. The present study only assessed the screening performance of various algorithms in GBM, and thus study results might not be extrapolatable to other populations at high risk of anal cancer. We only assessed these screening algorithms in their association with 12‐month persistent anal HSIL due to the study design, further follow‐up screening schedule beyond 12 months requires further study, as discussed in the consensus screening guidelines recommended by the International Anal Neoplasia Society.^14^

Even when persistent anal HSIL was used as the disease endpoint for anal cancer screening, the specificity of primary anal HRHPV testing (i.e., prevalent HPV16 infection or persistent non‐16 HRHPV infection) was low (<40%) and referral HRA rate was high (~70%). The number of referral HRAs needed would prevent it being used as a means of primary anal cancer screening even in resource‐rich countries like Australia, which has limited availability of qualified anoscopists who can perform high‐quality HRA. In addition, the high cost associated with HRA would make it unlikely to be cost‐effective to be publicly funded as a screening program.^41^

The triage use of anal cytology in GBM testing positive for non‐16HPV has the potential to improve the screening specificity and reduce the referral rate by ~20%, respectively, with a marginal loss of sensitivity by 5%. The size of the population at the highest risk for anal cancer is much smaller than that of women undergoing cervical cancer screening. Hence, the extra number of HRA referrals generated due to the lower specificity of a screening test will not be as overwhelming to the health system. Further research to establish more accurate HPV biomarkers, including methylation,^42^ will be required to further improve screening performance in anal cancer prevention.

## AUTHOR CONTRIBUTIONS

**Fengyi Jin:** Conceptualization; methodology; validation; formal analysis; writing—original draft; writing—review and editing; data curation. **I. Mary Poynten:** Conceptualization; writing—review and editing; project administration. **Richard J. Hillman:** Investigation; writing—review and editing. **Carmella Law:** Investigation; writing—review and editing. **Monica Molano:** Writing—review and editing; supervision. **Christopher K. Fairley:** Funding acquisition; writing—review and editing. **Suzanne M. Garland:** Funding acquisition; writing—review and editing. **David J. Templeton:** Investigation; writing—review and editing. **Andrew E. Grulich:** Conceptualization; funding acquisition; supervision; project administration; writing—review and editing. **Jennifer Roberts:** Conceptualization; methodology; validation; supervision; writing—review and editing.

## FUNDING INFORMATION

The Study of the Prevention of Anal Cancer is funded by a NHMRC program grant (number 568971) and a Cancer Council NSW Strategic Research Partnership Program grant (number 13‐11). Cytological testing materials are provided by Hologic (Australia) Pty Ltd. The Kirby Institute is affiliated with the Faculty of Medicine, University of New South Wales and funded by the Australian Government of Health and Ageing. The views expressed in this publication do not necessarily represent the position of the Australian Government.

## CONFLICT OF INTEREST STATEMENT

AEG has received honoraria and research funding from CSL Biotherapies and honoraria and travel funding from Merck. RJH has received honoraria and travel funding from CSL Biotherapies, and Merck. CKF owns shares in CSL. He has received support from CSL Biotherapies and MSD. SMG is the President of the International Papillomavirus Society, a consultant to Merck on HPV vaccines, and Working Group Member for HPV to SAGE, WHO. All other authors declare that they have no conflicts of interest.

## ETHICS STATEMENT

Ethics approval was granted by the Human Research Ethics Committees at the St Vincent's Hospital, Sydney and the University of New South Wales (HREC/09/SVH/168). Signed informed consent was obtained from all participants.

## Data Availability

All source code is publicly available on GitHub (https://github.com/jeffjin70/Coding-for-anal-cancer-screening-algorithm). The data that support the findings of this study are available from the corresponding author upon reasonable request.
